# Neonatal Pneumopericardium

**DOI:** 10.1016/j.atssr.2025.09.026

**Published:** 2025-10-23

**Authors:** Jiantao Zhang, Zheng Zhang

**Affiliations:** Department of Thoracic Surgery, The Affiliated Yantai Yuhuangding Hospital of Qingdao University, Shandong, China

A term female infant (37^+2^ weeks) delivered vaginally exhibited vigorous initial adaptation (Apgar 10 at 5 minutes). Acute respiratory decompensation occurred at 7 minutes, manifesting as distress, grunting, and pallor, reducing the Apgar score to 5 by 10 minutes, necessitating resuscitation and neonatal intensive care unit admission.

Admission findings included lethargy, ashen-grey color, tachypnea (62 breaths/min), coarse breath sounds, muffled heart sounds, hypotonia, diminished reflexes, and profound metabolic acidosis. Critical interventions within 30 minutes included mechanical ventilation, volume expansion, acidosis correction, and antibiotics.

**Figure 1:**
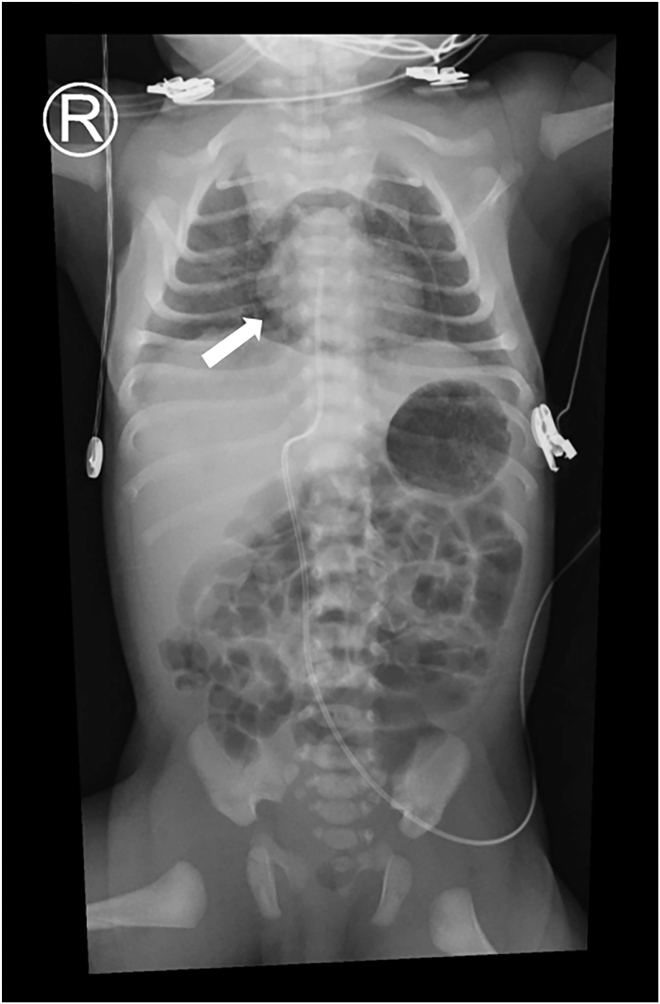

A 3-hour postnatal chest roentgenogram revealed pathognomonic pneumopericardium (Figure 1, arrow). It shows a continuous radiolucent rim (arrow) encircling the cardiac silhouette at 3 hours postnatal.

**Figure 2:**
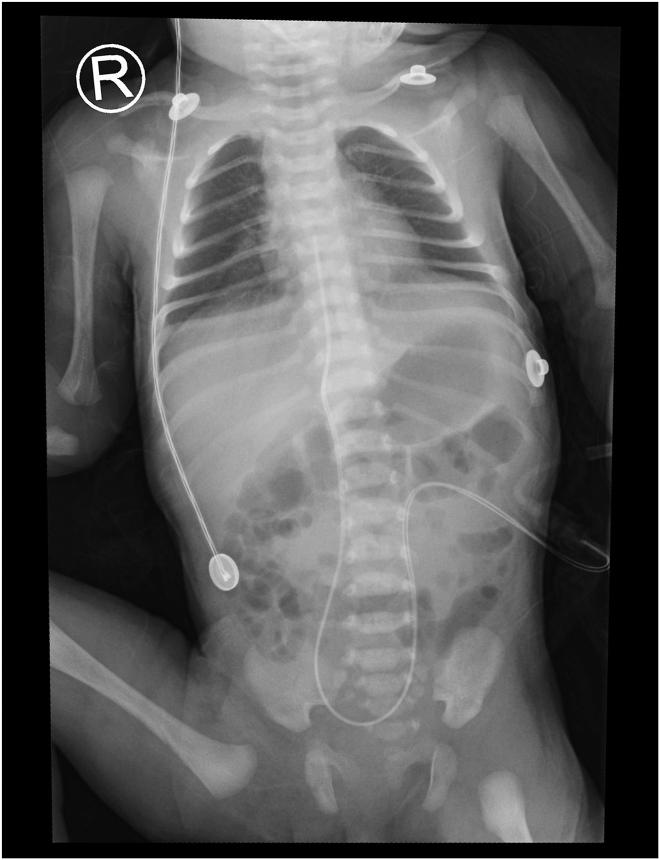

Pericardiocentesis was deferred. Clinical improvement enabled extubation at 10 hours. Serial radiographs showed complete spontaneous resolution of the pneumopericardium and concurrent pneumonitic changes by 40 hours (Figure 2). The infant recovered uneventfully and was discharged on day 7 and remained well with normal growth and development at the 1-month follow-up.

Neonatal pneumopericardium is exceedingly rare. This case is notable for the spontaneous occurrence of isolated pneumopericardium in a term neonate without antecedent trauma or identifiable risk factors beyond the initial respiratory distress. Crucially, the infant demonstrated rapid clinical stabilization and complete radiographic resolution with conservative management alone, avoiding invasive pericardial drainage. Based on rapid clinical improvement without tamponade signs, pericardiocentesis was deferred; it would be considered for hemodynamic compromise. Although idiopathic, a full workup includes echocardiography, computed tomography, and microbiology, but was not needed due to swift resolution.

